# Key challenges in bringing CRISPR-mediated somatic cell therapy into the clinic

**DOI:** 10.1186/s13073-017-0475-4

**Published:** 2017-09-25

**Authors:** Dianne Nicol, Lisa Eckstein, Michael Morrison, Jacob S. Sherkow, Margaret Otlowski, Tess Whitton, Tania Bubela, Kathryn P. Burdon, Don Chalmers, Sarah Chan, Jac Charlesworth, Christine Critchley, Merlin Crossley, Sheryl de Lacey, Joanne L. Dickinson, Alex W. Hewitt, Joanne Kamens, Kazuto Kato, Erika Kleiderman, Satoshi Kodama, John Liddicoat, David A. Mackey, Ainsley J. Newson, Jane Nielsen, Jennifer K. Wagner, Rebekah E. McWhirter

**Affiliations:** 1 Centre for Law and Genetics, Faculty of Law, University of Tasmania, Hobart, 7001 Australia; 2 Centre for Health, Law and Emerging Technologies, Nuffield Department of Population Health, University of Oxford, Oxford, OX2 7DD UK; 3 Innovation Center for Law and Technology, New York Law School, New York, 10013 USA; 4 Menzies Institute for Medical Research, University of Tasmania, Hobart, 7000 Australia; 5 Faculty of Health Sciences, Simon Fraser University, Burnaby, BC V5A1S6 Canada; 6 Usher Institute for Population Health Sciences and Informatics, University of Edinburgh, Edinburgh, EH8 9YL UK; 7 Department of Statistics, Data Science and Epidemiology, Swinburne University of Technology, Melbourne, 3122 Australia; 8 School of Biotechnology and Biomolecular Sciences, University of New South Wales, Sydney, 2052 Australia; 9 School of Nursing and Midwifery, Flinders University, Adelaide, 5042 Australia; 10 Addgene, Cambridge, 02139 USA; 11 Department of Biomedical Ethics and Public Policy, Graduate School of Medicine, Osaka University, Osaka, 565-0871 Japan; 12 Centre of Genomics and Policy, Department of Human Genetics, McGill University, Montreal, H3A0G4 Canada; 13 Department of Ethic, Graduate School of Letters, Kyoto University, Kyoto, 606-8501 Japan; 14 Faculty of Law, University of Cambridge, Cambridge, CB3 9DZ UK; 15 Centre for Ophthalmology and Visual Science, University of Western Australia, Lions Eye Institute, Perth, 6009 Australia; 16 Sydney Health Ethics, Sydney School of Public Health, University of Sydney, Sydney, 2006 Australia; 17 Center for Translational Bioethics & Health Care Policy, Geisinger Health System, Danville, PA 17822 USA

## Abstract

Genome editing using clustered regularly interspersed short palindromic repeats (CRISPR) and CRISPR-associated proteins offers the potential to facilitate safe and effective treatment of genetic diseases refractory to other types of intervention. Here, we identify some of the major challenges for clinicians, regulators, and human research ethics committees in the clinical translation of CRISPR-mediated somatic cell therapy.

## Regulatory challenges for CRISPR-mediated somatic cell therapy

The discovery that clustered regularly interspersed short palindromic repeats (CRISPR) and CRISPR-associated proteins found naturally in prokaryotic cells can be used to alter the genome of living organisms—including humans—has been one of the most exciting breakthroughs in biomedical science. Perhaps unsurprisingly, commentary on the ethical, social, and legal implications has focused primarily on concerns that, in the future, the technology could be used to heritably edit human germ cells, creating “designer babies”.

The current intense focus on germline genome editing—while it is understandable and necessary—diverts attention away from other pressing issues associated with clinical delivery in the non-germline context, where only the treated individual is affected. Given that attempts are already being made to use CRISPR-mediated somatic cell therapy to correct genetic mutations in diseases that are refractory to traditional therapies, somatic genome editing is an arguably more pressing issue [1].

Here we consider the regulatory conditions that need to be clarified for the medical community and the wider public to feel confident about moving somatic genome editing from bench to bedside. The regulatory parameters must be strong enough to engender confidence yet sufficiently flexible to respond to technological and social developments; a challenge that most jurisdictions are yet to fully address. We posit that these regulatory parameters should ideally be consistent globally, to provide greater certainty for the rapidly expanding industry and consistent safeguards for recipients. However, we recognize the almost insurmountable hurdles involved in achieving this end.

## Refocusing CRISPR law and ethics towards the clinic

There has been some progress towards identifying core principles to guide the responsible development and use of somatic genome editing. A report by the US National Academies [2] and a preliminary report by the UK Nuffield Council on Bioethics [3] both made important contributions in this regard. Notably, the US report concluded that current regulatory processes are adequate in the context of somatic cell therapies. However, this ignores the fact that CRISPR, in particular, and emergent personalized somatic cell therapies, in general, pose a range of challenges for regulators. Table 1 provides a non-exhaustive list of specific regulatory uncertainties that require attention; they are not necessarily new, nor are they specific to CRISPR. For instance, the first European ex vivo gene therapy was approved for marketing in 2016, following prolonged clinical trials starting in 2000 [4]. Many other gene and regenerative therapies are also currently under trial. The emergence and rapid uptake of CRISPR, and the promise it holds in the delivery of a broader range of somatic cell therapies, makes the discussion around the need to resolve regulatory uncertainities more pressing than ever.

**Table 1:** Issues in applying existing review and approval processes to clustered regularly interspersed short palindromic repeats (CRISPR)-mediated genome editing

Approval pathway	Issues to be resolved
Drugs and medical devices	Medical products generally go through some form of pre-market assessment to demonstrate clinical utility, safety, and efficacy. Many jurisdictions have developed adaptive licensing schemes including “fast track” approval, hospital exemptions, and compassionate use provisions that permit accelerated access to innovative treatments where they address unmet clinical need. These schemes can be invaluable in building up an early evidence base for promising but disruptive interventions, as many clinical CRISPR applications are likely to be, but at present there is little coordination across jurisdictions about the criteria to access these schemes or recognition of the evidence they generate.
Medical procedures	There is ambiguity over whether CRISPR-mediated genome-editing technology is characterized as a medical product or procedure. This means it is unclear whether pre-market approval is required or whether, like surgical procedures, early assessment should be governed by domestic professional societies and institutional funding.
Patient-tailored precision therapies	The high variability of biological manufacturing processes, where each batch can effectively be considered a separate product, and the individual patient-focused nature of many cell and gene therapies challenges the reliance on large-scale double-blind, placebo-controlled randomized controlled trials as the most relevant model for producing evidence of clinical safety and efficacy. A significant issue for CRISPR is whether patient-specific CRISPR constructs would each require separate approval or whether constructs with common characteristics can be treated as a group.
Exempted products	Not all medical products require regulatory approval. The exemption of human tissues and cells collected from patients and returned to them after ex vivo treatment is one example. This exemption could, at least theoretically, extend to certain CRISPR applications.
Public versus private funding	Different levels of protection may apply depending on whether the application is being developed by a publicly or privately funded institution. In the United States, for example, submission of gene therapy proposals to the recombinant DNA advisory committee (RAC) is only mandatory for research conducted at institutions receiving National Institutes of Health funding. For gene therapy at private institutions, submission to the RAC is voluntary. As public–private consortia become common tools for facilitating translational medicine, hard regulatory boundaries between public and private actors are likely to stifle innovation rather than secure safety.
Technology-specific regulation	Many nations have specific regulatory requirements for research involving transfer of genes (e.g., the RAC in the United States, and the Office of the Gene Technology Regulator in Australia). Designed to respond to technological capabilities as they existed at the point of enactment, these laws do not necessarily address the demands of fast-paced developments. In Europe, for example, there is confusion as to whether CRISPR-engineered organisms count as “genetically modified organisms”. Different European Union Member States classify clinical gene therapies as either “contained use” or “deliberate release” of a genetically modified organism, meaning the same procedure can be subject to different interpretations of the same European Union rules in different territories.

Responses to the regulatory uncertainties outlined in Table 1 need to be nuanced and flexible. The dangers of overly rigid regulatory responses to emerging technologies (e.g., through “command and control” legislation) have been demonstrated; for example, Canadian and Australian legislative responses to reproductive cloning, 15 years on, prevent scientists from undertaking work on some forms of mitochondrial transfer in those countries, irrespective of the potential benefits. Given the difficulty in future-proofing, mechanisms for periodic review need to be included to ensure responsiveness to evolving understanding of novel technologies and re-evaluation of their harms and benefits. This may help to prevent prematurely locking in regulatory parameters, which can otherwise remain untouched for generations, as has happened with the US Common Rule [5]. In the context of genome editing, and of any other emergent personalized therapy, regulation must be sufficiently flexible and technologically adaptive to address new technological advances and applications as they arise.

## Broader stakeholder engagement in risk assessment

Quantifying the likely harms and benefits of novel, disruptive therapeutics such as genome editing will always involve considerable uncertainty. Within the permissible spectrum of CRISPR-mediated activities, rigorous regulatory approaches for risk and benefit assessment will be essential. This is not to assert that all regulatory insufficiencies require probing evaluations of their specific applicability and adequacy in respect of genome editing. Jurisdictions may legitimately set different thresholds for an acceptable risk–benefit ratio. Nonetheless, several features of any assessment process should be universally required. In particular, regulatory processes must be grounded in adequate scientific expertise, but take into account social as well as technical aspects of risk.

We argue that it will be challenging for clinicians or regulatory agencies to navigate the risks and benefits of translating genome editing into the clinic by relying on expert judgement alone, and that broader consultation is required. Trials of CRISPR-mediated somatic cell therapies present an opportunity to engage a wider range of stakeholders, especially patients and patient groups, in determining the acceptable thresholds for risk and benefit. Ideally, this means not only considering what magnitude of risk is acceptable, but discussing which outcomes are taken into consideration, and what counts as harm or benefit, and to whom [6].

Regulators have traditionally shown reluctance to engage widely in the course of their risk–benefit assessments. National regulators of medical products, for example, often operate in conditions of secrecy, with the European Medicines Agency, the Japanese Pharmaceuticals and Medical Devices Agency, and the Australian Therapeutic Good Administration typically only publishing information about product reviews and the evidence on which they rest after they have made made their decision. By contrast, US Food and Drug Administration Advisory Committee meetings are open to public participation.

Human research ethics committees, which take responsibility for approving and monitoring clinical trials in most countries, present a greater opportunity for broad engagement. They are generally required to have a diverse membership base, including members who are not trained in science. Nonetheless, medical and scientific members have consistently been assessed as having a greater influence on committee decision-making than others, even where community-wide ethical issues are present [7]. And astonishingly, some research ethics codes (e.g., the US Common Rule [5]) expressly prohibit considering the potential long-term social implications of a research project.

Some programmes, such as the European Patients’ Academy on Therapeutic Innovation and the US Professional Patient Advocate Institute provide training and support to enable patients to engage as advocates in the drug discovery process. But it remains unclear whether there will be longer-term institutional support for more direct involvement of patients and their representatives [8]. Incorporating improved public and stakeholder engagement mechanisms will assist in future-proofing regulation. More work needs to be done to develop appropriate mechanisms for open and inclusive multi-stakeholder dialogue.

## Financial and intellectual property imperatives

Another complication in the further clinical development of CRISPR technology is the need to ensure that the financial benefits of clinical delivery outweigh the costs.

One way to reduce the large financial burdens associated with medical product reviews and approvals is to harmonize processes across countries. This means that if an approval is gained in one country, the same evidence can be used for approval in another (and perhaps even be automatically granted). Such harmonization would permit product development companies to reach economies of scale more quickly, reducing the cost of medical products and resulting in more favorable cost–benefit analyses. Although international harmonization is difficult to achieve, regulators should continue to work towards this goal, and considerable efforts have already been made in this regard.

Reimbursement is also a significant consideration. There are different national systems for evaluating whether a given intervention has adequate cost–benefit returns to make it worth paying for. This, when conducted by bodies like the UK National Institute for Health and Clinical Excellence, is sometimes regarded as the “fourth hurdle” of regulation. Glybera, the first gene therapy product to get European marketing authorization and approval, has recently been discontinued by its manufacturers following poor uptake by physicians resulting from cost recovery issues [9].

Patent rights add yet another layer of complexity. The development of any new technology generally prompts a raft of patent filings. In the case of genome editing this has been accompanied by a prolonged patent dispute, which has implications for both commercial and clinical applications. To date, this dispute has been resolved differently in Europe and the United States, although further judicial proceedings are likely in both jurisdictions.

Issues surrounding patent ownership and validity feed into clinical delivery. After all, a product will only be developed and marketed if there is some prospect of recouping research and development costs. The temporary exclusivity provided by patents can facilitate this. Patents can also be used as tools to minimize social harm and maximize social benefit if owners are able to negotiate ethical terms into patent license agreements. By contrast, delegation by owners to a small number of surrogate licensors—who may themselves be in competition with other developers—has the potential to create bottlenecks in rapidly developing fields [10]. In addition, patent licenses that incorporate significantly different terms for public and private end users may cause problems for academic–industry partnerships working to develop innovative CRISPR therapeutics. Ambiguities in the patent landscape surrounding CRISPR are likely to further confuse clinical translation pathways.

## Conclusion

New technologies—and CRISPR-mediated genome editing in particular—often elicit a plethora of ethical, medical, and commercial questions from a variety of stakeholders, raising regulatory and social challenges. This is the case both in areas where there are overlapping regulatory obligations (regulatory congestions) and in areas where there is an absence of regulation (regulatory gaps) [11]. But focusing on any technology’s extremes—especially where “worst case scenarios” are unrealistic or technologically impractical—often results in rigid, top-down approaches, prone to be reactive and piecemeal. Focusing on regulatory issues in somatic—rather than germline—genome editing applications is of more immediate benefit to patients and highlights the gaps and congestions in most urgent need of attention.

Here, we have identified key areas of regulatory confusion that are likely to impede the process of translating somatic genome editing to the clinic. Resolving these issues will encourage the development and approval of applications that are both clinically robust—safe, efficient, and for the benefit of patients—and socially robust—accountable, democratic, and trustworthy.

## Abbreviations

CRISPR: Clustered regularly interspersed short palindromic repeats
